# Supplementary material for: Denosumab Is Superior to Raloxifene in Lowering Risks of Mortality and Ischemic Stroke in Osteoporotic Women
Source: Pharmaceuticals (Basel). 2023 Feb 1;16(2):222. doi: 10.3390/ph16020222 (PMC9966982; doi:10.3390/ph16020222)
Supplement: Supplementary file 1 [file pharmaceuticals-16-00222-s001.zip › pharmaceuticals-2104525-supplementary.pdf]

Table S1. Codes for inclusion/exclusion criteria, baseline comorbid conditions and medication use.

Inclusion/exclusion criteria and baseline comorbidity	
Myocardial infarction	ICD-9-CM: 410-414<br>ICD-10-CM: I21, I22, I24, I25
Congestive heart failure	ICD-9-CM: 428<br>ICD-10-CM: I5020-I5023, I5030-I5033, I5040-I5043, I509, I501
Ischemic stroke	ICD-9-CM: 433-437<br>ICD-10-CM: I63, I65, I66, I67.0~I67.2, I67.4~I67.82, I67.841~I67.9, I68, G45.0~G45.2, G45.4~G45.9, G46
*Peripheral vascular diseases	ICD-9-CM: 441, 443.9, 785.4, V434<br>ICD-10-CM: I71, I73, I79
*Cerebral vascular accident	ICD-9-CM: 430, 431, 432, 433, 434, 435, 436, 437, 438<br>ICD-10-CM: I60, I61, I62, I63, I65, I66, I67, I69, G45, G46
*Dementia	ICD-9-CM: 290<br>ICD-10-CM: F01, F02, F05
*Pulmonary disease	ICD-9-CM: 490, 491, 492, 493, 494, 495, 496, 500, 501, 502, 503, 504, 505<br>ICD-10-CM: J40, J41, J42, J44, J43, J44, J45, J47, J60, J61, J62, J63, J64, J65, J66, J67
*Connective tissue disorder	ICD-9-CM: 517.1, 710, 714, 725<br>ICD-10-CM: M05, M06, M32, M33, M34, M35
*Peptic ulcer	ICD-9-CM: 531, 532, 533, 534<br>ICD-10-CM: K25, K26, K27, K28
*Liver diseases	ICD-9-CM: 571<br>ICD-10-CM: K70, K71, K73, K74
*Diabetes	ICD-9-CM: 250.0, 250.1, 250.2<br>ICD-10-CM: E10.1, E10.9, E11.0, E11.9, E13.1, E13.9
*Diabetes complications	ICD-9-CM: 250.4, 250.5, 250.6, 250.7, 250.8, 250.9<br>ICD-10-CM: E10.2, E10.3, E10.4, E10.5, E10.6, E10.8, E11.2, E11.3, E11.4, E11.5, E11.6, E11.8, E13.2, E13.3, E13.4, E13.5, E13.6, E13.8
*Paraplegia	ICD-9-CM: 342, 344.1<br>ICD-10-CM: G04.1, G81, G82
*Renal disease	ICD-9-CM: 582, 583, 585, 586, 588<br>ICD-10-CM: N01, N03, N05, N07, N18, N19, N25
*Severe liver diseases	ICD-9-CM: 572.2, 572.3, 572.4, 572.8<br>ICD-10-CM: K72.1, K72.9, K76.6, K76.7
Hypertension	ICD-9-CM: 401, 402, 403, 404, 405<br>ICD-10-CM: I10, I11, I12, I13, I15, N26.2
Hyperlipidemia	ICD-9-CM: 272<br>ICD-10-CM: E75.2, E75.3, E75.5, E75.6, E77, E78, E88.1, E88.2, E88.89
Thyroid function abnormal	ICD-9-CM: 242, 243, 244<br>ICD-10-CM: E00, E01, E03, E05, E89.0
Obstructive sleep apnea	ICD-9-CM: 780.51, 780.53, 780.57

	ICD-10-CM: G47.30, G47.33, G47.39
Cancer	ICD-9-CM: 140-208<br>ICD-10-CM: Cxxx, Z51
Kidney transplantation	ICD-9-CM: V42.0<br>ICD-10-CM: Z94<br>**Billing code: 76020A, 76020B
End stage Kidney disease	. Hemodialysis:<br>ICD-9-CM/PCS: 39.95<br>ICD-10-PCS: 5A1D60Z, 5A1D00Z<br>**Billing code: 58001C, 58002CB, 58014C, 58018C, 58019C, 58020C, 58021C, 58022C, 58023C, 58024C, 58025C, 58027C, 58029C, 58030B<br>. Peritoneal dialysis:<br>ICD-9-CM/PCS: 54.98<br>ICD-10-PCS: 3E1M39Z<br>**Billing code: 58002C, 58009B, 58010A, 58010B, 58011A, 58011AB, 58017B
**Baseline medications**	**Anatomical Therapeutic Chemical (ATC) code**
Denosumab	M05BX04
Raloxifene	G03XC01
Oral anticoagulants	B01AA, B01AE, B01AF
Anti-platelet	B01AC
Aspirin	B01AC06
Statins	C10AA, C10BA, C10BX
Fibrates	C10AB, C10BA
Other lipid-lowering agents	C10AC, C10AX
Anti-diabetics	A10
ACEI/ARB/Aliskiren	C09AA, C09BB, C09CA, C09DA, C09DB, C09DX, C09XA
Bisphosphonates	M05BA02, M05BA03, M05BA06, M05BA07, M05BA08
Alendronate	M05BA04, M05BB03
Teriparatide	H05AA02
Calcitonin preparations	H05BA01, H05BA02, H05BA03
Calcium	A12AA04, A12AA12, A12AA91, A12AX
Vitamin D	A12AX, A11CC07

Prior use of medication was identified <365 days before the index date with > 28 days of supply
Concomitant use of medication was identified between the index date and the earliest date of event of interest with > 28 days of supply. ICD-9-CM/PCS: International Classification of Diseases, Ninth Revision, Clinical Modification and Procedure Classification System; ICD-10-CM/PCS: International Classification of Diseases, Tenth Revision, Clinical Modification and Procedure Classification System.
